# Familial Glucocorticoid Deficiency Type 2: A Case Report

**DOI:** 10.4274/jcrpe.v2i3.122

**Published:** 2010-08-06

**Authors:** Leyla Akın, Selim Kurtoğlu, Mustafa Kendirici, Mustafa Ali Akın

**Affiliations:** 1 Erciyes University, Faculty of Medicine Department of Pediatric Endocrinology, Kayseri, Turkey; 2 Erciyes University, Faculty of Medicine Department of Neonatology, Kayseri, Turkey; +90 352 438 00 76leylabakin@gmail.comErciyes University Faculty of Medicine Department of Pediatrics 55139, Kurupelit, Kayseri, Turkey

**Keywords:** Familial glucocorticoid deficiency, ACTH unresponsiveness, MRAP

## Abstract

Familial glucocorticoid deficiency (FGD) is a rare autosomal recessive disease resulting from resistance to the action of adrenocorticotropic hormone (ACTH) on the adrenal cortex, which leads to isolated glucocorticoid deficiency with normal mineralocorticoid secretion. It may present in infancy or early childhood with hyperpigmentation, failure to thrive, recurrent infections, hypoglycemic attacks and convulsions that may result in coma or death. Laboratory investigations reveal low cortisol and androgen levels with high ACTH associated with normal reninaldosterone axis. The disorder may be caused by mutations in the gene of ACTH receptor (MC2R), or mutations in the newly described melanocortin− 2 receptor accessory protein (MRAP) namely, FGD type 1 and FGD type 2, respectively. Twenty five percent of FGD cases are due to the mutations of the ACTH receptor, while FGD type 2 accounts for approximately 15−20% of FGD cases. Here, we report a six−month−old male infant, who presented with recurrent hypoglycemic convulsions. Serum hormone analysis showed low cortisol and androgen levels associated with a high ACTH concentration. No mutation was found in the NR0B1 and MC2R genes excluding congenital adrenal hypoplasia and FGD type 1. We found a homozygous deletion (c. 106+1delG) in intron 3 of MRAP gene. To our knowledge, this is the first Turkish patient reported with FGD type 2 due to a known MRAP mutation.

**Conflict of interest:**None declared.

## INTRODUCTION

Familial glucocorticoid deficiency (FGD), or hereditary unresponsiveness to adrenocorticotropic hormone (ACTH), is a rare autosomal recessive disease characterized by glucocorticoid deficiency in the absence of mineralocorticoid deficiency. Mutations of the ACTH receptor, also known as the melanocortin−2 receptor (MC2R), account for approximately 25% of FGD cases (1). More recently, Metherell et al (2) demonstrated that mutations in melanocortin−2 receptor accessory protein (MRAP), encoding a new interacting partner of the ACTH receptor, caused FGD in 19 of 104 kindreds with confirmed FGD and no ACTH receptor mutations, and they account for a further 15–20% of FGD cases.

In the current paper, we describe the first Turkish patient with FGD type 2 due to a known MRAP mutation.

## CASE REPORT

**Figure 1:**
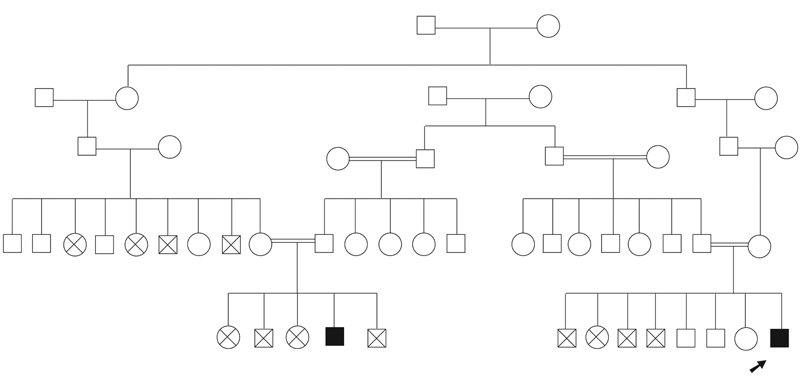
Family pedigree showing the relationship of the patient to the affected cousin and also to the unexplained neonatal deaths in his siblings and relatives.

A male baby was referred to our clinic for evaluation of hypoglycemic convulsions at the age of six months. He was born to consanguineous parents after an uneventful pregnancy with a birth weight of 4750 g. Medical history revealed that he had had a convulsion on the first day of life and had been receiving antiepileptic treatment since then. While on antiepileptic treatment, he had convulsive episodes again on three different occasions. The etiology of the convulsions had not been evaluated before. He was the eighth child of the family. Four of his siblings, three males and one female, had died on the first day of their lives. The other three siblings, two males and a female, were reported to be healthy. The baby had a male cousin, who was diagnosed as a case of isolated cortisol deficiency (Figure 1).

On physical examination, length was 73 cm (97^th^ percentile; SDS:+2), weight 8700 g (90^th^ percentile; SDS:+1.4), and head circumference was 46 cm (97^th^ percentile). Blood pressure was 90/60 mmHg. His midparental target height was 167 cm (10^th^ percentile, SDS:−1). Examination of the external genitalia revealed a penis of normal length and bilaterally palpable testes in the scrotum. Hyperpigmentation of the skin was noted. There were no signs of alacrima or achalasia. Other examination findings were unremarkable. Blood chemistry results were: glucose: 63 mg/dL, Na: 140 mmol/L, K: 4 mmol/L, Cl: 109 mmol/L, ALT: 28 IU/mL, and AST: 30 IU/mL. Total blood count was normal. Serum hormone analyses showed the following: 17−OH progesterone: 0.01 ng/mL (0.03−0.9), androstenedione: 0.01 ng/mL (<0.1−0.17), DHEA−S: 3 ng/mL (50−480), cortisol: 0.6 μg/dL (2.8−23), ACTH:708 pg/mL (6−48), plasma renin activity (PRA): 57 ng/mL/hr (2.35−37), aldosterone: 801 pg/mL (50−900). The bone age, evaluated by X−ray of the left hand and wrist according to the Greulich−Pyle atlas, was found to be equivalent to 9 months. Adrenal ultrasonography revealed that both adrenal glands were small in size −left: 6x8 mm, and right: 7x8 mm in diameter. Cranial ultrasonography was normal. Based on these findings, a diagnosis of isolated glucocorticoid deficiency was made, and oral hydrocortisone treatment at a dose of 10 mg/m^2^/d was started.

For the molecular diagnosis, the entire coding region of the NR0B1 (DAX1), the MC2R gene and the 6 exons of the MRAP gene were amplified by PCR with intronic primers, and the PCR products on both the sense and antisense strand were sequenced. No mutation was found in the NR0B1 and MC2R genes. We found a homozygous deletion of one nucleotide at the canonical 5α donor splice site (c.106+1delG) in intron 3 of MRAP gene. The mutation was confirmed with a second independent PCR and sequence reaction. The mother was found to be a heterozygous carrier for the 1−bp deletion in intron 3.

On his most recent visit, at the age of four years and eight months, the patient had no hyperpigmentation. His height was 103 cm (25^th^ percentile), weight 17 kg (25^th^ percentile), testis volumes bilaterally were 2 ml with a penile length of 5 cm. The bone age was evaluated as 4 years. His neurodevelopment was appropriate for age. The ACTH level was suppressed to slightly above the normal limit (53 pg/ml) by 20 mg/m^2^/d oral hydrocortisone treatment.

An informed consent for publication was taken from the mother.

## DISCUSSION

FGD is a rare autosomal recessive disorder characterized by isolated glucocorticoid deficiency in the absence of mineralocorticoid deficiency. These patients have low cortisol and high ACTH levels with a normal renin−aldosterone axis. FGD usually presents in infancy or in early childhood with hyperpigmentation, failure to thrive, recurrent infections, hypoglycemic attacks and convulsions that may result in coma or death (3).

The differential diagnosis of adrenal insufficiency in infancy includes congenital disorders such as congenital adrenal hyperplasia, adrenal hypoplasia, drenoleukodystrophy

(ALD), ACTH resistance syndromes (FGD, Allgrove syndrome) and Kearns−Sayre syndrome; and acquired conditions such as adrenal hemorrhage, trauma and infections (4, 5). Congenital adrenal hyperplasia was excluded by hormone analysis; acquired causes of adrenal insufficiency were also excluded by history and laboratory findings. Allgrove syndrome was not considered because of the absence of alacrima or achalasia. ACTH receptor gene sequences were normal in our patient excluding FGD type 1. Congenital adrenal hypoplasia was also unlikely, because he had well−developed genitalia and no mutation of the DAX1 gene.

Mutations of ACTH receptor (MC2R) account for about 25% of FGD cases (FGD type 1, OMIM 202200) (1). Recently, mutations in MRAP gene, a gene encoding a small single transmembrane domain protein known as MRAP, have been described in a group of patients with ACTH resistance syndrome but with no mutations in MC2R gene (FGD type 2, OMIM 607398). MRAP, located at 21q22.1, is an essential cofactor for MC2R expression in certain cell types and seems to have a role in the processing, trafficking, or function of MC2R (2). MRAP has two isoforms, namely, MRAPα and MRAPβ which differentially regulate the function of MC2R (6), and so far 9 different mutations of MRAP in FGD patients have been described; they all result in either an absent or significantly truncated protein of both isoforms (7). MRAP mutations comprise approximately 20% of patients with FGD (2). In our patient, we found a 1−bp deletion at the canonical 5α donor splice site (c.106+1delG) in intron 3 of MRAP gene in the DNA of the patient in a homozygous state. His mother was heterozygous for the 1−bp deletion. In their study, Metherell et al (2) identified the 1−bp deletion, c. 106+1delG, in 6 individuals from 5 families with glucocorticoid deficiency, making this the second frequent mutation causing FGD unrelated to defects in the MC2R gene. To our current knowledge, 1−bp deletion (c. 106+1delG) in intron 3 of MRAP gene, identified in the DNA of the patient, can be regarded as the cause of FGD type 2.

In the only study regarding molecular diagnosis of FGD patients from Turkey, Berberoglu et al (8) reported 5 patients with FGD type 1. In this series, three siblings had homozygous V 142 L mutations and the other two siblings had homozygous D103N mutations in MC2R gene. To our knowledge, our patient is the first reported Turkish patient with FGD type 2, with a known MRAP mutation. The patient was the offspring of consanguineous parents and his four siblings had died in the neonatal period, probably due to glucocorticoid insufficiency.

The long−term neurological consequences of FGD depend on the severity and number of hypoglycemic episodes during childhood. Modan−Moses et al (9) reported an Ethiopian infant, who presented with psychomotor retardation, spastic quadriparesis and microcephaly due to severe hypoglycemic attacks. They speculated that this phenotype could result from a novel MRAP mutation. Our patient was diagnosed with FGD at the age of six months and had four convulsion episodes until presentation. Despite this history of several hypoglycemic attacks, at his most recent visit at age of four years and eight months, the patient’s neurodevelopment was appropriate for age.

Patients with FGD often have undetectable levels of adrenal androgens (10). In our patient also, 17−OH progesterone, androstenedione and DHEA−S levels were very low.

Tall stature is observed in some FGD type 1 cases with MC2R mutations (11). Bone age was reported to be advanced in some of these patients, a finding, which was more pronounced before the initiation of treatment. It is reported that hydrocortisone replacement may decrease the height growth. Nevertheless, these children become tall adults (12). The excessive growth has been proposed to be due to high plasma ACTH levels (13). A possible mechanism for excessive growth is that all five melanocortin receptors are present in bone, and ACTH can stimulate cAMP production and gene expression in bone cells (11, 14).

Unlike FGD type1, FGD type 2 patients are known to be of normal height. At presentation, our patient’s length, weight, and head circumference percentiles were all above the parental target. Bone age was also advanced by three months. Although length in infancy may not reflect final height, we suggest that increased ACTH or related pathophysiologic changes might have caused advanced growth in this patient. After the initiation of treatment, anthropometric percentiles gradually decreased and came down to 25th percentile at age four years and eight months. On the most recent visit, it was observed that the bone age was delayed by about 8 months. The patient will be monitored closely for the effects of possible overdose of treatment.

FGD is characterized by isolated glucocorticoid deficiency in the presence of a normal renin−aldosterone axis. However, mild derangement of the renin−angiotensin system and mild salt−wasting at the time of diagnosis have been reported in some cases (12, 15). In our patient, the initial PRA was also moderately elevated despite normal serum electrolyte levels. However, PRA and aldosterone levels on follow−up have remained within normal ranges. In these patients, definitive diagnosis can be made by finding mutations in the MC2R or recently described MRAP gene. Nevertheless, in about 55−60% of the FGD cases, the gene defect causing the disease has remained unidentified.

In conclusion, clinical awareness of this condition is of considerable prognostic and therapeutic significance. Further studies describing new cases and mutations causing FGD will contribute to understanding the mechanism of this rare and potentially life−threatening disease.

## ACKNOWLEDGEMENT

The authors would like to thank Dr. Tim Strom (Institute of Human Genetics, Technical University Munich, Germany) for performing the genetic analyses on our patient and his mother)
